# Ultrasound data for laboratory calibration of an analytical model to calculate crack depth on asphalt pavements

**DOI:** 10.1016/j.dib.2017.06.053

**Published:** 2017-07-05

**Authors:** Miguel A. Franesqui, Jorge Yepes, Cándida García-González

**Affiliations:** a Grupo de Fabricación Integral y Avanzada − Departamento de Ingeniería Civil, Universidad de Las Palmas de Gran Canaria (ULPGC), Campus de Tafira 35017 Las Palmas de Gran Canaria, Spain; b Departamento de Ingeniería Civil − IOCAG, Universidad de Las Palmas de Gran Canaria (ULPGC), Campus de Tafira 35017 Las Palmas de Gran Canaria, Spain

**Keywords:** Pavement cracking, Crack depth, Surface-breaking crack, Top-down cracking (TDC), Non-destructive testing, Ultrasound, Pavement maintenance, Volcanic aggregate

## Abstract

This article outlines the ultrasound data employed to calibrate in the laboratory an analytical model that permits the calculation of the depth of partial-depth surface-initiated cracks on bituminous pavements using this non-destructive technique. This initial calibration is required so that the model provides sufficient precision during practical application. The ultrasonic pulse transit times were measured on beam samples of different asphalt mixtures (semi-dense asphalt concrete AC-S; asphalt concrete for very thin layers BBTM; and porous asphalt PA). The cracks on the laboratory samples were simulated by means of notches of variable depths. With the data of ultrasound transmission time ratios, curve-fittings were carried out on the analytical model, thus determining the regression parameters and their statistical dispersion. The calibrated models obtained from laboratory datasets were subsequently applied to auscultate the evolution of the crack depth after microwaves exposure in the research article entitled “Top-down cracking self-healing of asphalt pavements with steel filler from industrial waste applying microwaves” (Franesqui et al., 2017) [1].

**Specifications Table**

**Table:** 

Subject area	*Civil Engineering*
More specific subject area	*Pavement Engineering and Maintenance*
Type of data	*Text file, Tables, Figures*
How data was acquired	*Ultrasonic Pulse Velocity (UPV) measurements based on time-of-flight diffraction (TOFD). Ultrasound device: CSI type CCT-4 “concrete tester”, cylindrical couplant plate contact (CPC) transducers, 26* *mm diameter, 54 kHz. T=20 °C.*
Data format	*Averaged experimental measurements*
Experimental factors	*3 types of hot mix asphalt (HMA): AC-S, BBTM, PA. Crack depths: 10 to 50* *mm. Temperature: 20 °C. Ultrasound frequency: 54 kHz.*
Experimental features	*Slab specimens cut in prismatic beam samples. Cracks simulated by means of notches (4–5* *mm slot). Samples previously conditioned at 20 °C (4 hours). 10 repeated ultrasonic measurements on each sample under the same experimental conditions (material, sample, crack depth).*
Data source location	*Laboratory of Highway Engineering, Department of Civil Engineering, University of Las Palmas de Gran Canaria (ULPGC), 35017 Las Palmas de Gran Canaria, Canary Islands, Spain, 28°4’11.669”N; 15°27’19.843”W*
Data accessibility	*Data is with this article*

**Value of the data**

• The data allows verification of the model´s precision and the statistical dispersion of the ultrasound measurements on 3 types of asphalt mixes (AC-S, BBTM and PA). It also becomes a starting point for future research regarding other types of mixtures. Up until now ultrasound transmission time ratios on different types of bituminous mixtures have not been systematically reported.
• It will enable the comparison with data obtained in future research using other ultrasonic transducers (dry point contact [DPC] sensors, different pulse frequencies and baselines).
• The data has facilitated to obtain empirical recommendations for the practical implementation of this non-destructive technique using ultrasounds on asphalt mixtures: pulse frequency and baseline selection, and minimum measurement requirements.

## 1 Data

The dataset within this article provides averaged values of ultrasonic pulse transit time and velocity measurements (Table 3) and transmission ratios (Table 4) on prismatic laboratory samples of different bituminous mixtures: semi-dense asphalt concrete (AC-S), gap-graded asphalt concrete for very thin layers (AC-TL, also known in Europe as BBTM) and porous asphalt (PA). All of these materials are commonly used in surface layers of asphalt pavements. By means of regression analysis, the aforementioned data was used to calibrate an analytical model in order to determine the depth of a surface-breaking crack on asphalt pavements using ultrasounds (Eq. 6).

## 2 Materials

Slab specimens (300×300×60 mm) of 3 different types of hot mix asphalt (HMA) were prepared: a) semi-dense asphalt concrete (AC 16 surf 50/70 S); b) gap-graded asphalt concrete for very thin layers (BBTM 11B PMB 25/55–65); c) porous asphalt mixture (PA 11 PMB 25/55–65). Given that ultrasonic techniques are non-destructive, these specimens were subsequently used to monitor the self-healing and the evolution of the crack depth after repeated heating cycles applying microwaves (see Ref. [1]). For this reason, a steel filing with corumdum powder of size less than 0.063 mm (90% steel / 10% corumdum, approximately, obtained from radial saw grindings) was used to substitute the mineral filler (metallic filler). The three different aforementioned asphalt mixtures were produced following the Spanish road specifications (PG-3) [2] (in particular, aggregate gradation for each type of mixture, minimum bitumen content and air void content) and compacted by rolling according to EN 12697-33. The main properties of the mixtures and the component materials are summarized in Table 1. Additional characterization properties of the mixtures can be seen in Ref. [1].

**Table 1:** Main properties of the different types of HMA and the component materials used for the laboratory samples.

					AC 16 surf 50/70 S (EN 13108-1)		BBTM 11B PMB 25/55–65 (EN 13108-2)		PA 11 PMB 25/55–65 (EN 13108-7)
Aggregate	Type of aggregate (all the fractions)				Massive phonolite (volcanic rock)		Massive phonolite (volcanic rock)		Massive phonolite (volcanic rock)
	Particle density (g/cm^3^) EN 1097-6: ρ_a_ / ρ_rd_ / ρ_SSD_		# 10–20 mm		2.65 / 2.56 / 2.58		2.65 / 2.56 / 2.58		2.65 / 2.56 / 2.58
			# 4–10 mm		2.67 / 2.48 / 2.52		2.67 / 2.48 / 2.52		2.67 / 2.48 / 2.52
			# 0–4 mm		2.67 / 2.50 / 2.57		2.67 / 2.50 / 2.57		2.67 / 2.50 / 2.57
	WA_24_ (%)				2.10		2.10		2.10
	LA coefficient EN 1097-2				19		19		19
	M_DE_ coefficient EN 1097-1				15		15		15
Bitumen	(%) (by total wt. of mixture)				4.5		5.0		4.5
	Type of bitumen				Penetration bitumen		Polymer modified		Polymer modified
	Penetration at 25 °C (x0.1 mm) EN 1426				44		37		37
	Softening Point (°C) EN 1427				52		67		67
Metallic filler [# <0.063 mm] (%)					5.37		5.50		4.50
Volumetric properties of the mixture		Maximum density (g/cm^3^) EN 12697-5			2.55		2.54		2.56
		Bulk density (g/cm^3^) EN 12697-6			2.40		2.06		1.94
		Void content (%) EN 12697-8			5.75		18.63		24.32
Performance characteristics		Sm_[0.6]_ (MPa) EN 12697-26 [IT-CY test at 20 °C]		5625		3750		3830	

(ρ_a_) Particle density [apparent]; (ρ_rd_) Particle density [dry]; (ρ_SSD_) Particle density [saturated surface dry]; (WA_24_) Water absorption after 24 hours; (LA) Resistance to fragmentation of the aggregate [Los Angeles coefficient]; (M_DE_) Resistance to wear [micro-Deval coefficient]; (Sm_[0.6]_) Stiffness modulus from indirect tensile test on cylindrical specimens with a load surface factor (k) of 0.6 at a temperature of 20 °C

## 3 Instruments

The ultrasonic device was a CSI type CCT-4 “concrete tester” (resolution ±0.1 µs) with cylindrical couplant plate contact (CPC) piezoelectric transducers of 26 mm diameter and 54 kHz. Despite the fact that high-frequency ultrasounds present better beam directivity, defined onset, sensitivity and lateral resolution characteristics, high energy pulses and relatively low excitation frequencies are necessary when working with bituminous mixtures (inhomogeneous and viscoelastic materials) due to the high wave attenuation caused by absorptive and scattering phenomena as well as to the limited size of the aggregates and cracks being investigated. Ultrasonic testing on Portland cement concrete, for instance, is usually restricted to frequencies of less than 150 kHz [3] to avoid considerable levels of coherent (microstructure) noise. The frequencies employed during ultrasonic testing should decrease as crack depth and temperature increase to compensate for elastodynamic wave scattering. According to the experience obtained from data in Ref. [1], working frequencies for bituminous mixtures should be less than 70 kHz, recommending if possible transducers with a frequency between 24 and 54 kHz, being low frequencies more suitable for very long path lengths and greater maximum size of aggregate.

A heater-cooler was employed to condition temperature of test samples, with a capacity of 150 l, forced air circulation, range 4 to 65 °C and resolution ±0.1 °C.

## 4 Methodology

### 4.1 Procedure and analytical model

**Fig. 1:**
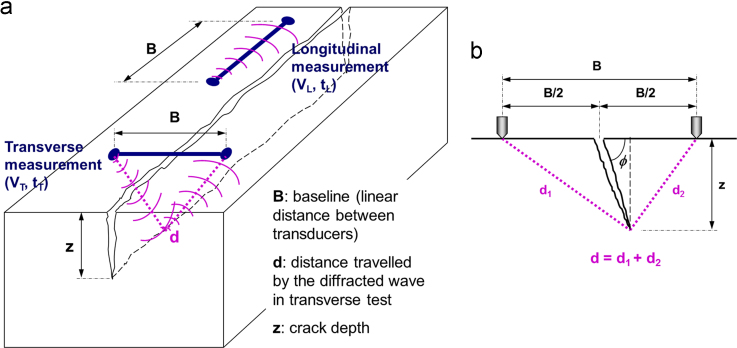
a) Diagram of longitudinal and transverse ultrasound measurements with a surface-breaking crack; b) Assumed theoretical wave propagation model in transverse test.

The ultrasonic pulse velocity (UPV) analysis in the time domain (times-of-flight and velocities of transient elastic waves) was employed. However, an auto-calibration procedure for practical applications is required in order to eliminate the need for preliminary calibration and prior knowledge of the mechanical properties or wave velocity of the materials being tested. As pavement can only be accessed on its surface, it was utilized a simplified, one-sided (indirect), self-compensating wave transmission scheme involving surface and body waves, as suggested by Ref. [4]. It incorporates ultrasonic transmission measurements that are longitudinal and transverse to the crack (Fig. 1a). As all measurements are expressed as a ratio, this auto-calibration technique eliminates the effects of those factors and errors that influence both tests equally and also the need for previous knowledge of the elastic properties of the material being investigated.

In order to demonstrate the final model we have proposed in Eq. (6), a geometric acoustics postulate based on the linear ray propagation of waves in a semi-infinite space is assumed. According to Fig. 1a, as the distance between probes in both measurements remains constant:

(1) $$\begin{array}{c} V_{L}=B/t_{L}\\ V_{T}=B/t_{T} \end{array}\}\to \frac{V_{T}}{V_{L}}=\frac{t_{L}}{t_{T}}$$

Where: *B* = baseline (linear distance between transducers or sensing points); *V*_*L*_, *V*_*T*_ = pulse velocity in longitudinal and transverse measurements, respectively; *t*_*L*_, *t*_*T*_ = time-of-flight in longitudinal and transverse measurements, respectively.

Note that *V*_*T*_ is an apparent velocity and *V*_*L*_ is a true velocity in the longitudinal test. Under the same temperature conditions, the latter velocity is constant in the material in all directions, assuming isotropy of the material in a limited layer thickness. Thus, it can easily be demonstrated that the ratios between distances travelled in both tests are directly proportional to the time ratios (or inversely proportional to the apparent velocity ratios):

(2) $$\frac{B}{d}=\frac{V_{T}}{V_{L}}=\frac{t_{L}}{t_{T}}$$

Where: *d* = distance travelled by the diffracted ultrasonic pulse in the transverse test.

Based on the above-mentioned simplified model of linear wavefront propagation in homogeneous and isotropic media, and on the geometric model in Fig. 1b, a relationship between the distance travelled *d* and the crack depth *z* (for a crack with a dip angle *ϕ*) can be algebraically calculated:

(3) $$d=d_{1}+d_{2}=\sqrt{z^{2}+{\left(\frac{B}{2}+\frac{z}{tg\phi }\right)}^{2}}+\sqrt{z^{2}+{\left(\frac{B}{2}-\frac{z}{tg\phi }\right)}^{2}}$$

By combining Eqs. (2), (3), the depth *z* of a surface-breaking crack can be estimated using the time-of-flight or velocity ratios, providing the following approximate theoretical transmission model:

(4) $$\frac{V_{T}}{V_{L}}=\frac{t_{L}}{t_{T}}=\frac{B}{\sqrt{z^{2}+{\left(\frac{B}{2}+\frac{z}{tg\phi }\right)}^{2}}+\sqrt{z^{2}+{\left(\frac{B}{2}-\frac{z}{tg\phi }\right)}^{2}}}$$

With discontinuities (notches, cracks) being completely vertical (1/tg*ϕ*=0), this equation can be simplified to:

(5) $$\frac{V_{T}}{V_{L}}=\frac{t_{L}}{t_{T}}=\frac{B}{\sqrt{B^{2}+4\cdot z^{2}}}=\frac{1}{\sqrt{1+4\cdot {\left(z/B\right)}^{2}}}$$

### 4.2 Preparation of laboratory samples and ultrasonic measurements

**Fig. 2:**
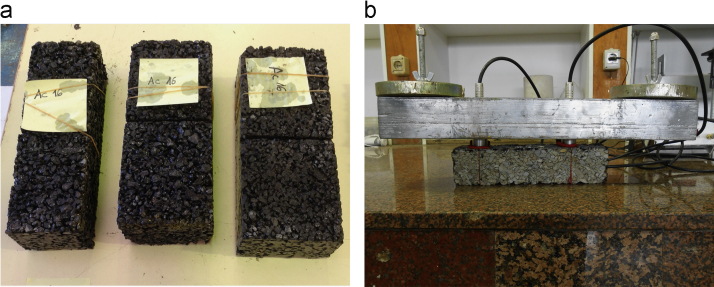
a) Prismatic samples of AC16 S notched to simulate in laboratory a partial-depth surface-initiated crack; b) Example of the arrangement to obtain transit time and pulse velocity data applying a one-sided (or indirect) ultrasound transmission scheme.

By cutting the slab specimens (300×300×60 mm), 9 prismatic beam samples were obtained (3 for each type of HMA); two of the beams measured 300×110×60 mm and the third measured 300×80×60 mm (for each HMA) (see Fig. 2a). Furthermore, to ensure independence of the ultrasonic pulse velocity regardless of the size and shape of the samples, the condition (*λ/w*)<1 (*λ*: ultrasound wavelength in each material at a certain temperature; *w*: least lateral dimension of the sample) was previously verified. Above this value, the transmission time may increase noticeably. The cracks for the calibration of the models were artificially simulated in the laboratory with notches made by a radial saw in the centre of the prismatic beams (notch depths: 10±1 [BBTM and PA], 20±1 [AC-S], 35±1 and 50±1 mm, with a 4–5 mm slot between notch faces). The prismatic beam samples were conditioned in a heater-refrigerator at 20 °C during 4 hours before the ultrasound measurements. Thus, all the models were obtained for this working temperature.

According to the analysis of the standard deviations of all ultrasound measurements recorded (Table 2), a minimum of nine repeated measurements under the same experimental conditions (statistical sample size) would be necessary in order to limit random errors (assuming a maximum error of 65% of the average standard deviation registered for one of these materials [0.33 µs], with a confidence level of 95%). Furthermore, practical experience based on ultrasound data with asphalt mixtures mentioned in Ref. [1] recommends a limited application of the models to ratio values (*z/B*)<0.3 to 0.4, so as to reduce measurement dispersion with ultrasounds, measurement errors and model errors due to a possible tilt of the cracks. Therefore, proportionality should be maintained between the crack depth (*z*) and the baseline (*B*) being used.

**Table 2:** Average standard deviation and coefficient of variation of ultrasound measurements on laboratory samples at 20 °C.

Type of HMA	Average standard deviation (*Sd*)	Average coefficient of variation (C_V_)
AC 16 surf 50/70S	0.28 μs	0.67%
BBTM 11B PMB 25/55-65	0.24 μs	0.76%
PA 11 PMB 25/55-65	0.33 μs	0.91%

Table 3 summarizes experimental data of ultrasonic pulse propagation times and velocities for the three types of HMA at 20 °C measured on laboratory prismatic samples with several baselines and crack depths. Table 4 shows data for the transmission time ratios at the same temperature.

**Table 3:** Ultrasonic pulse transit time and velocity for the three types of HMA at 20 °C.

**Type of HMA**	**B (mm)**	**z (mm)**	**t (µs)**	**V (m/s)**	**V**_**av**_**. (m/s)**	**λ**_**av**_**. (m)**
AC 16 surf 50/70 S	70	0	23.2	3017.24	2837.53	5.25·10^−2^
	80	0	24.9	3207.70		
	100	0	34.8	2876.87		
	120	0	43.9	2734.11		
	150	0	58.2	2575.55		
	200	0	76.5	2613.70		
	70	20	36.3	1931.03	1931.03	3.57·10^−2^
	120	35	61.2	1960.14	1960.14	3.63·10^−2^
	150	50	75.4	1990.18	1990.18	3.69·10^−2^
BBTM 11B PMB 25/55-65	70	0	21.2	3301.89	2880.71	5.76·10^−2^
	80	0	22.3	3585.84		
	100	0	33.4	2995.81		
	120	0	43.5	2759.26		
	150	0	59.2	2533.78		
	200	0	94.9	2107.70		
	70	10	27.6	2538.99	2538.99	4.70·10^−2^
	120	35	56.2	2136.75	2136.75	3.96·10^−2^
	150	50	78.1	1921.84	1921.84	3.56·10^−2^
PA 11 PMB 25/55-65	70	0	22.1	3174.60	2337.78	4.68·10^−2^
	80	0	31.1	2574.83		
	100	0	41.2	2426.60		
	120	0	50.7	2368.27		
	150	0	91.3	1643.30		
	200	0	108.8	1839.08		
	70	10	31.8	2203.34	2203.34	4.08·10^−2^
	120	35	83.3	1440.75	1440.75	2.67·10^−2^
	150	50	106.5	1408.32	1408.32	2.61·10^−2^

(B) Baseline; (z) Crack depth; (t) Ultrasound propagation time for a given crack depth; (V) Ultrasonic pulse velocity; (V_av._) Averaged ultrasonic pulse velocity; (λ_av._) Averaged ultrasound wavelength

**Table 4:** Experimental data of averaged transmission ratios for the three types of HMA at 20 °C.

Type of HMA	B (mm)	z (mm)	z/λ	(*t*_L_/*t*_T_) = (V_T_/*V*_L_)
AC 16 surf 50/70 S	70	0	0.00	1.00
		20	0.33	0.63
		30	0.49	0.49
	120	0	0.00	1.00
		35	0.64	0.73
		40	0.73	0.68
	150	0	0.00	1.00
		40	0.77	0.83
		50	0.97	0.77
BBTM 11B PMB 25/55-65	70	0	0.00	1.00
		10	0.15	0.80
		30	0.45	0.44
	120	0	0.00	1.00
		35	0.63	0.77
		40	0.72	0.72
	150	0	0.00	1.00
		40	0.79	0.81
		50	0.74	0.82
PA 11 PMB 25/55-65	70	0	0.00	1.00
		10	0.16	0.75
		30	0.47	0.36
	120	0	0.00	1.00
		35	0.73	0.60
		40	0.85	0.55
	150	0	0.00	1.00
		40	1.21	0.90
		50	1.52	0.86

(B) Baseline; (z) Crack depth; (z/λ) Normalized crack depth; (t_L_/t_T._) = (V_T_/V_L._) Transmission ratio

### 4.3 Calibrated model for the transmission ratio

The former simplified analytical model shown in Eq. (5) was expressed for normalized crack depths (*z/λ*), normalizing the independent variable. Therefore, the obtained models can be used for different wavelengths (*λ*) of pulse (different transducer frequencies, according to Section 3). In order to calibrate this model a regression curve was fitted to the experimental data points summarized on Table 4, applying a least squared method using the Levenberg-Marquardt iterative algorithm which allows for the minimization of a nonlinear function over a space of parameters. The regression model was satisfactorily approximated by a one-parameter function f[*z/(λB)*, *m*_*1*_] (see Eq. 6).

(6) $$\frac{t_{L}}{t_{T}}=\frac{B/\lambda }{\sqrt{{\left(B/\lambda \right)}^{2}+m_{1}\cdot {\left(z/\lambda \right)}^{2}}}$$

Fig. 2 in Ref. [1] shows the final functions of the calibrated model of each HMA, obtained for T=20 °C with measurement baseline (*B*) of 70, 120 and 150 mm. The coefficient of determination (R^2^), which represents the goodness-of-fit, ranged between 0.9638 and 0.9999. The good adjustment of the empirical data to this model means that the self-calibration measurement scheme successfully compensates for the effects of the elastic properties of the material, meaning that these properties need not be previously known in order to obtain valid transmission ratios. The method also eliminates the disrupting effects caused by coupling variability and internal and surface heterogeneities of the asphalt mixtures. The regression parameter (m_1_) for the best-fit curves and the coefficient of determination (R^2^) at a temperature of 20 °C can be consulted in Table 2 of Ref. [1].

These calibrated models were subsequently used to calculate the depth of the cracks on another series of prismatic beam samples of the same types of materials, and following different microwave exposure intervals in order to track the self-healing process of the cracks artificially produced in the laboratory (see Ref. [1]).
